# Supplementary material for: Sodium Pre-Intercalation-Based Na3-δ-MnO2@CC for High-Performance Aqueous Asymmetric Supercapacitor: Joint Experimental and DFT Study
Source: Nanomaterials (Basel). 2022 Aug 18;12(16):2856. doi: 10.3390/nano12162856 (PMC9414395; doi:10.3390/nano12162856)
Supplement: Supplementary file 1 [file nanomaterials-12-02856-s001.zip › nanomaterials-1747188-supplementary.pdf]

nanomaterials

Sodium Pre-Intercalation-Based Na_3-δ-MnO_2@CC for High-Performance Aqueous Asymmetric Supercapacitor: Joint Experimental and DFT Study

Anis Ur Rahman [1], Nighat Zarshad [2], Wu Jianghua [3], Muslim Shah [4], Sana Ullah [4], Guigen Li [1,5,*], Muhammad Tariq [6] and Asad Ali [4,*]

1 Institute of Chemistry and BioMedical Sciences, School of Chemistry and Chemical Engineering, Nanjing University, Nanjing 210023, China
2 Department of Polymer Science, School of Chemistry and Chemical Engineering, Southeast University, Nanjing 211189, China
3 National Laboratory of Solid State Microstructures, Collaborative Innovation Center of Advanced Microstructures and College of Engineering and Applied Sciences, Nanjing University, Nanjing 210093, China
4 Department of Chemistry, Faculty of Chemical and Life Sciences, Abdul Wali Khan University, Mardan KPK, 23200, Pakistan
5 Department of Chemistry and Biochemistry, Texas Tech University, Lubbock, TX 79409, USA
6 Department of PCB, Bayazid Rokhan Institute of Higher Studies, Kabul Jalalabad 1002, Afghanistan
* Correspondence: guigen.li@ttu.edu (G.L); asadali@awkum.edu.pk (A.A.)

The mass balance of cathode and anode is obtained from the following charge balance formula,

$$\frac{m_+}{m_-} = \frac{C_- \times \Delta E_-}{C_+ \times \Delta E_+} \quad (S1)$$

In above equation
m is the mass of the cathode and anode.
C is the specific capacitance.
ΔE (V) is the potential window of a single electrode and the subscript + and − are cathode and anode respectively.
The specific capacitance of the electrode was calculated by Equation (S2).

$$C = \frac{I\Delta t}{m\Delta V} \quad (S2)$$

Where
I (A) is the discharge current.
Δt (s) is the discharge time
m (g) is the mass of the active material.
ΔV (V) is the potential window of the discharge.
The energy density (E, Wh kg^{-1}) and power density (P, W kg^{-1}) of the asymmetric supercapacitor can be obtained by Equations (S3) and (S4).

$$E = \frac{1}{2}\frac{C(\Delta V)^2}{3.6} \quad (S3)$$

$$P = \frac{E_D \times 3600}{\Delta t} \quad (S4)$$

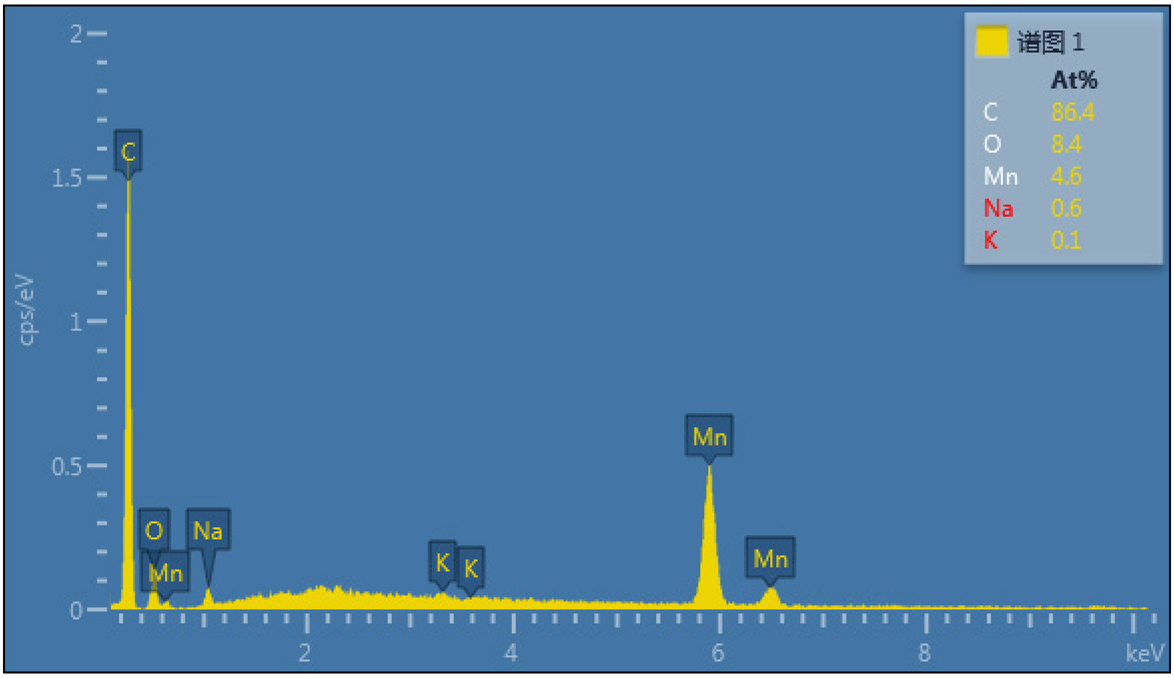

Figure S1. EDS elemental mapping of Na_3-MnO_2.

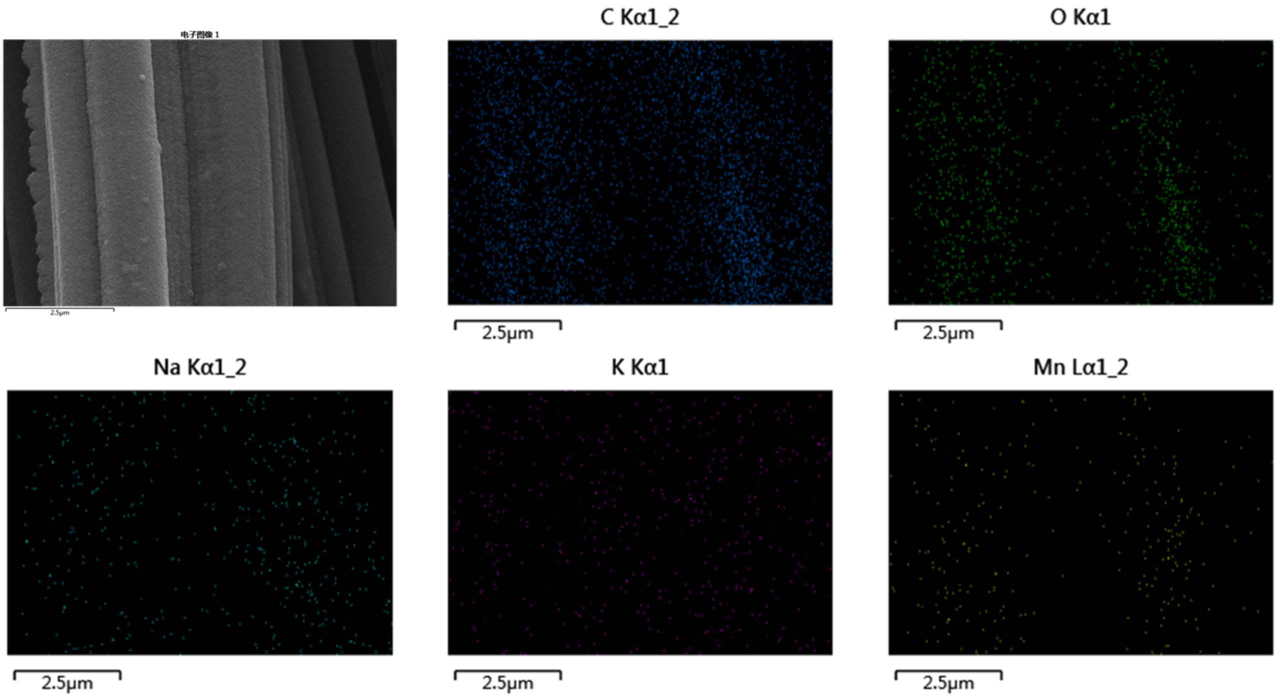

Figure S2. EDS mapping of Na_3-MnO_2.

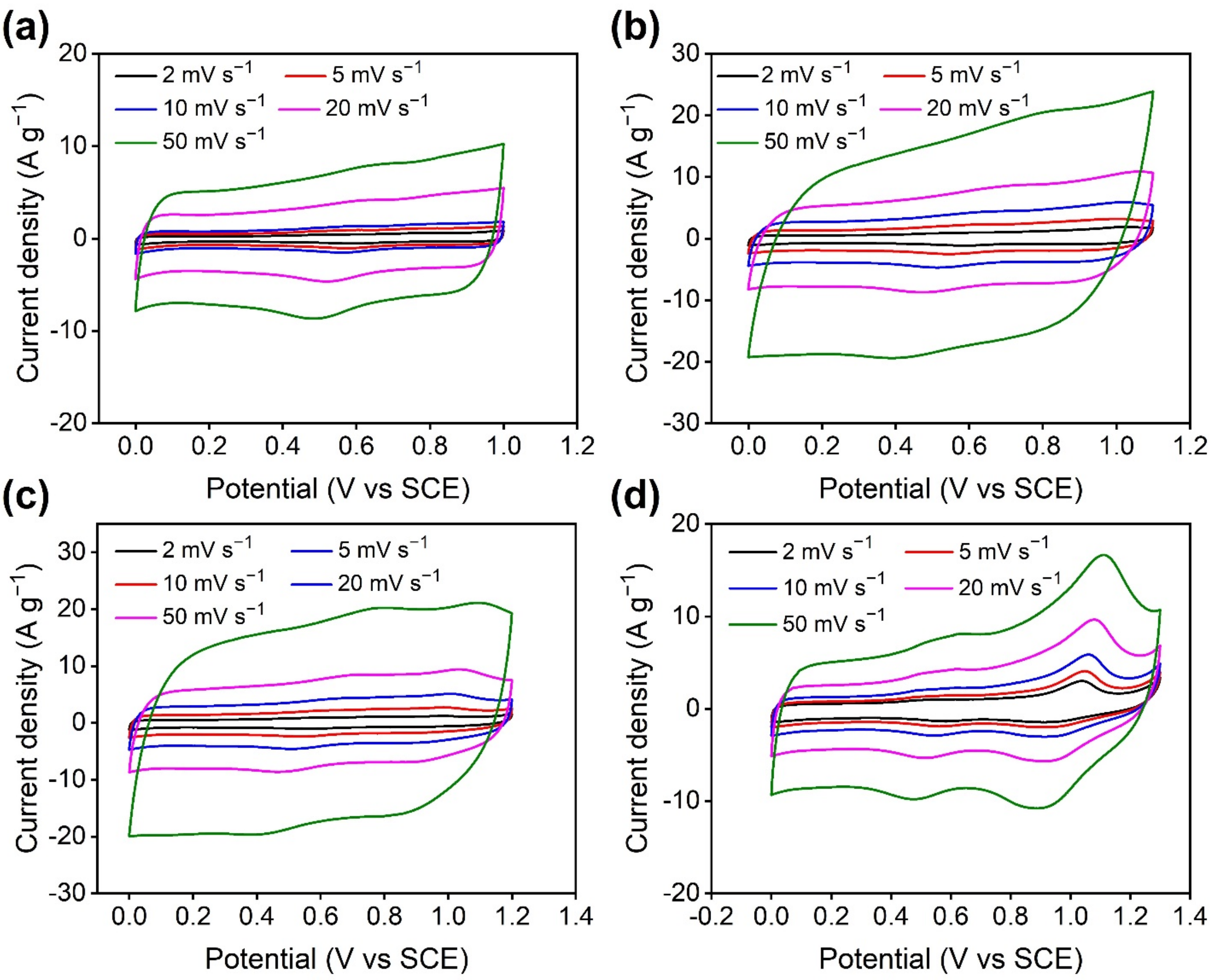

Figure S3. The CV of Na3-MnO2 at a scan rate of 1, 2, 5, 10, 20, and 50 mV s^{-1} in different working potential windows of (a) 0-1.0 V (b) 0-1.1V; (c) 0-1.2 V; and (d) 0-1.3 V.

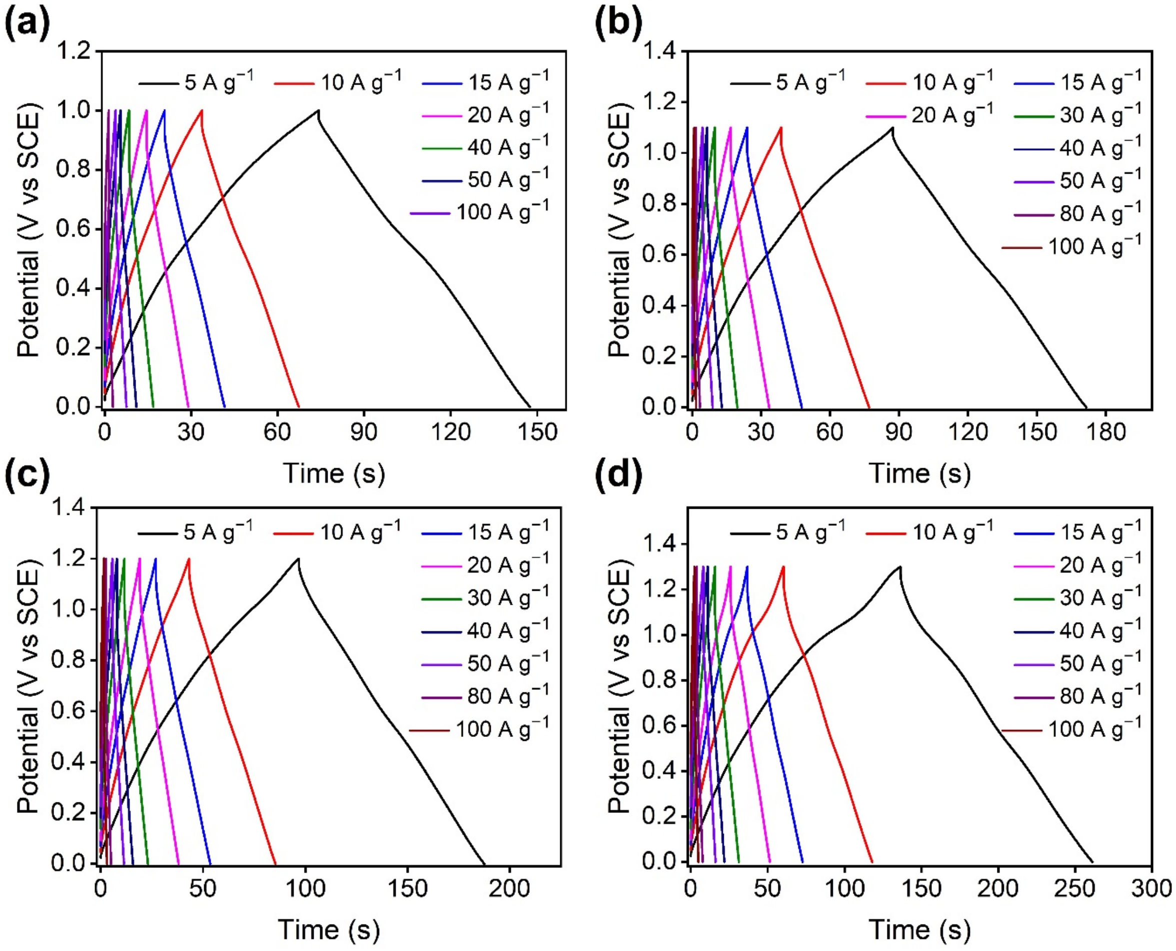

Figure S4. The GCD of Na3-MnO2 at various current densities of 5, 10, 15, 20, 30, 40, 50, 80 and 100 A g^{-1} in different working potential windows of (a) 0-1.0 V (b) 0-1.1V; (c) 0-1.2 V; and (d) 0-1.3 V.

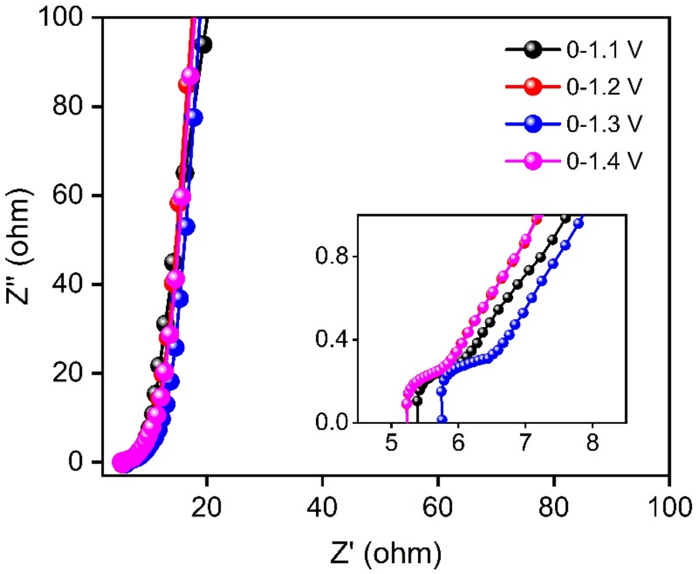

Figure S5. Nyquist plots recorded at 100 kHz to 10 MHz of Na3-MnO2 at different working potential windows.

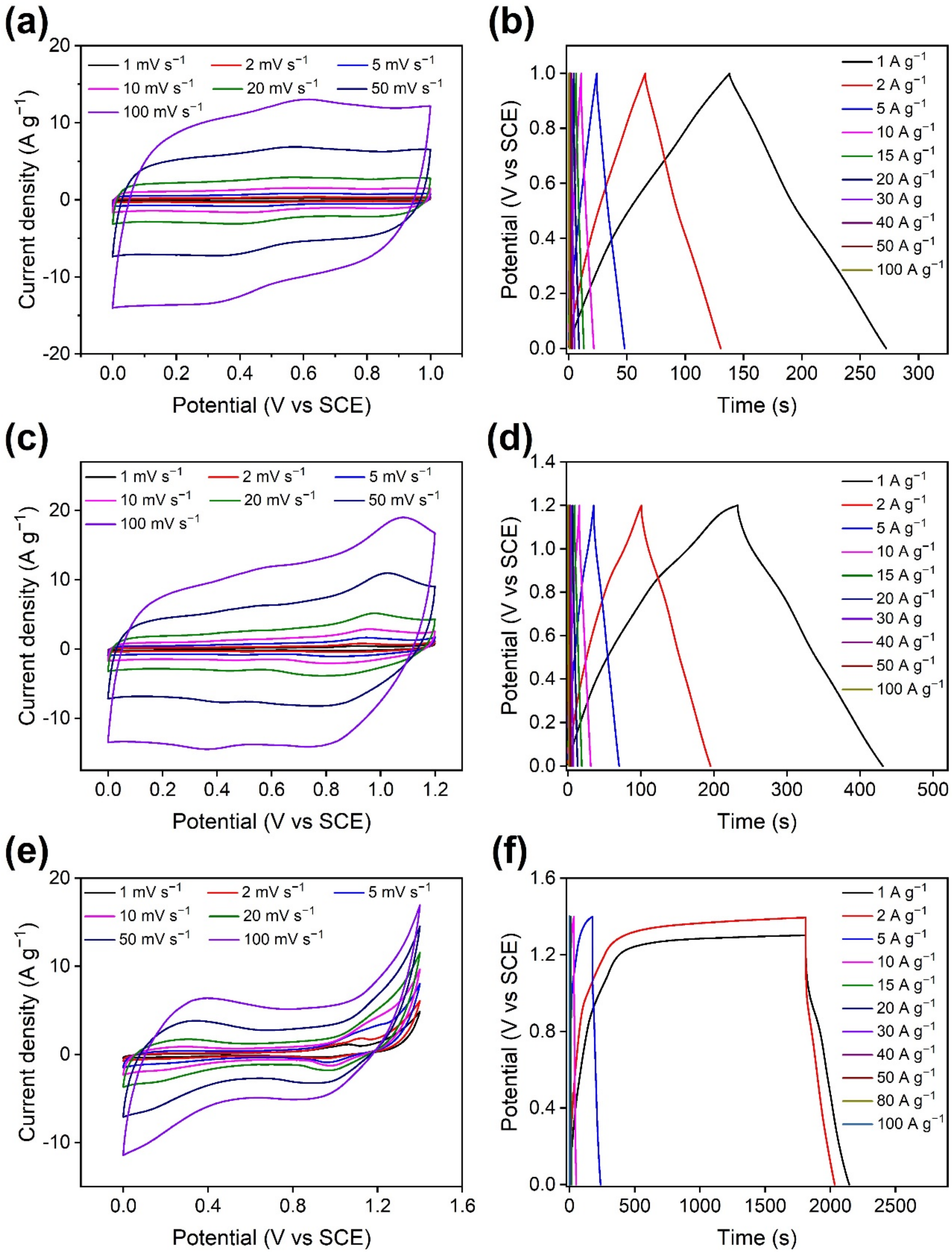

Figure S6. Electrochemical performance of the of MnO_2 without Na ions; CV at a scan rate of 1, 2, 5, 10, 20, 50 and 100 mV s^{-1} in different working potential windows (a) 0-1.0 V; (c) 0-1.2 V; (e) 0-1.4 V; GCD curves at a various current density of 1, 2, 5, 10, 15, 20, 30 ,40 50, 80 and 100 A g^{-1} in different working potential windows of (b) 0-1.0 V; (d) 0-1.2 V and (f) 0-1.4 V.

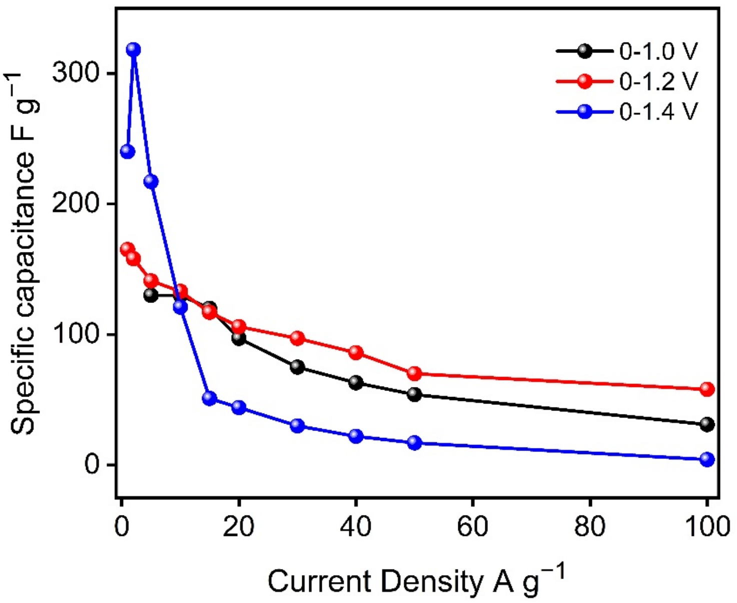

Figure S7. comparison of specific capacitance of MnO_2 electrode at different potential window of 0-1.0, 0-1.2, and 0-1.4 V as function of current density.

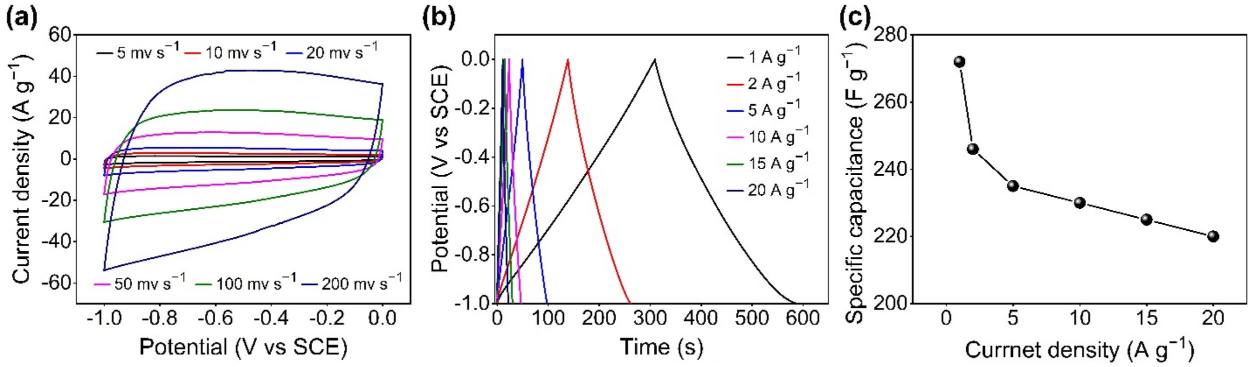

Figure S8. the electrochemical performance of AC was recorded in the three-electrode system; (a) CV of the at a scan rate of 10-200 mV s^{-1} in 0-1.0 V; (b) GC of the at a current density of 1-20 A g^{-1} in 0-1.0 V; (c) specific capacitance as a function of current density.

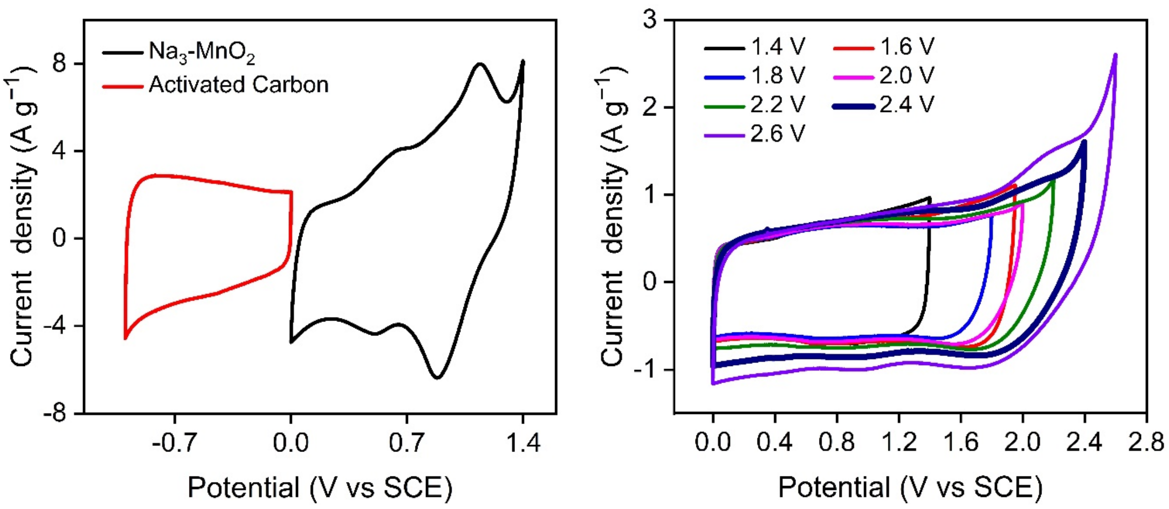

Figure S9. Comparison of CV curves of Na_3-MnO_2 electrode and N-AC in their separate potential windows of 1.4-0 V and 0-1.0 V respectively at a scan rate of 10 mV s^{-1}; (b) CV curves at different potential windows from 1.6 to 2.6 V were recorded at a scan rate of 10 mV s^{-1}.

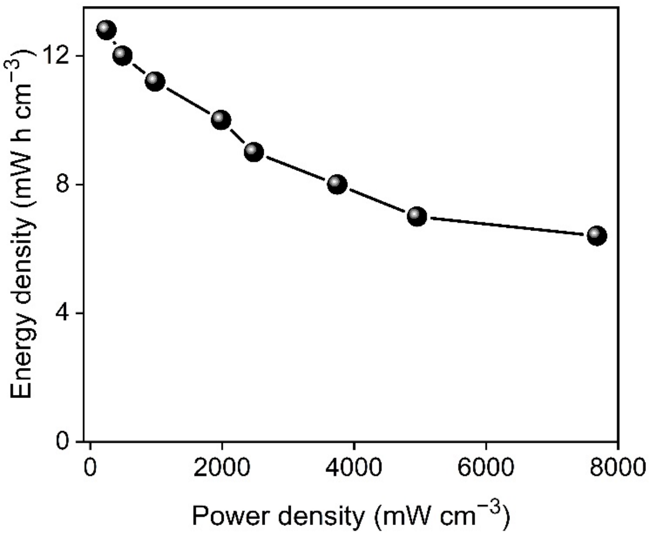

Figure S10. Ragone plots of Na_3-MnO_2 //N-AC device volumetric energy and power density.

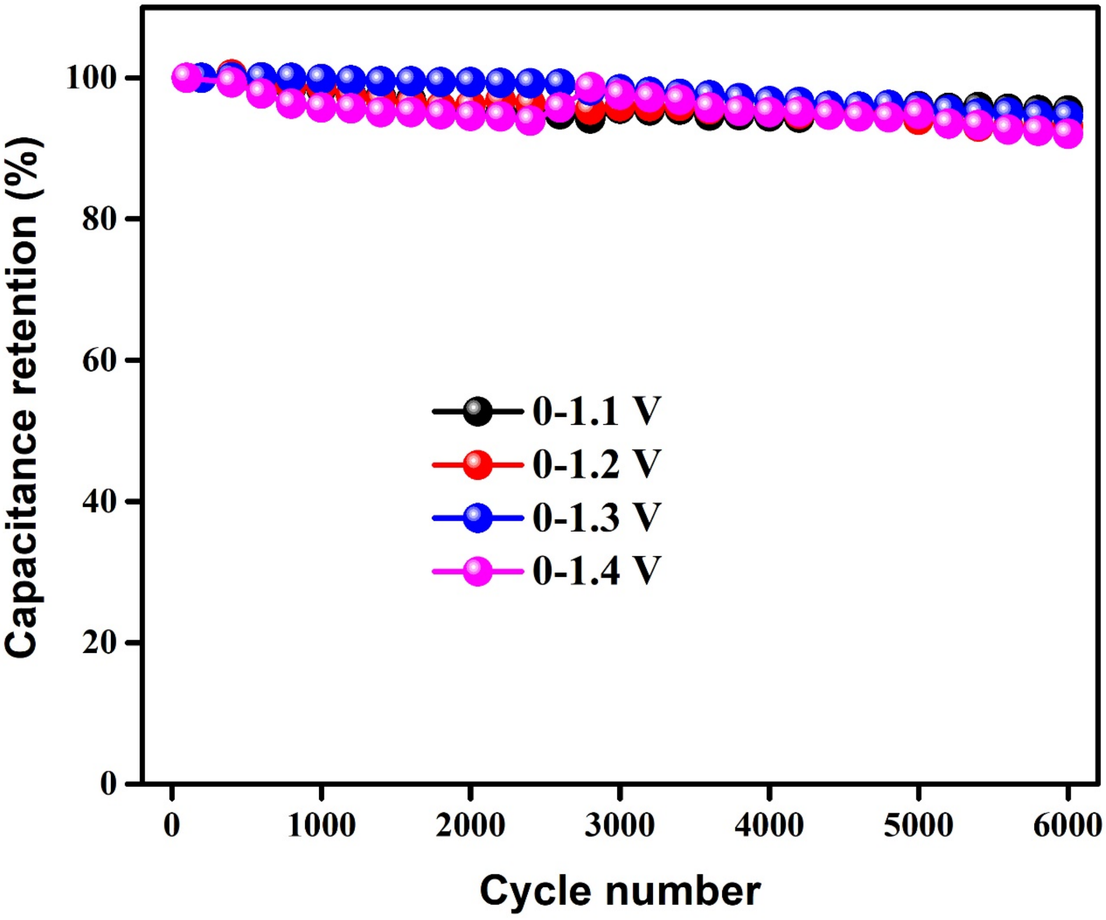

Figure S11. Cycling performance comparison at potential window of 0-1.0, 0-1.1, 0-1.2, 0-1.3, and 0-1.4 V.
